# A cross-sectional look at patient concerns in the first six weeks following primary total knee arthroplasty

**DOI:** 10.1186/1477-7525-5-48

**Published:** 2007-08-01

**Authors:** Ravi Rastogi, Aileen M Davis, Bert M Chesworth

**Affiliations:** 1 Physiotherapist, London Health Sciences Centre, London, Ontario, Canada; 2 Senior Scientist, Health Care and Outcomes Research Division and Arthritis Community Research Evaluation Unit, Toronto Western Research Institute; Associate Professor, University of Toronto Toronto, Ontario, Canada; 3 Assistant Professor, School of Physical Therapy and Bachelor of Health Sciences Program, Faculty of Health Sciences and Department of Epidemiology and Biostatistics, Schulich School of Medicine and Dentistry, The University of Western Ontario, London, Ontario, Canada

## Abstract

**Background:**

To date, no researchers have investigated patient concerns in the first six weeks following primary total knee arthroplasty (TKA). An understanding of patient concerns at a time when physical therapists are involved in the treatment of these patients will aid clinicians in providing patient-centered care. Linking of items to the International Classification of Functioning, Disability and Health (ICF) allows for comparison and sharing of data amongst researchers, as the ICF is the accepted framework for evaluating disability in rehabilitation. The objective of this study was to identify patient concerns in the first six weeks following primary TKA and link these concerns to components of the ICF and map them to commonly used outcome measures.

**Methods:**

Individual interviews were conducted to identify patient concerns during their recovery following primary TKA. Concerns identified by patients were analysed for content and linked to the components of the ICF using the operational definitions of the ICF components. These concerns were mapped to the WOMAC, KOOS and Oxford Knee Scale.

**Results:**

Thirty patients (18 female) with an average age (SD) of 68.4 (11.1) years completed the study. Patients identified 32 concerns. Twenty-two percent (n = 7) of the concerns linked to Body Function and Structure, 47% (n = 15) to Activity, 13% (n = 4) to Participation, and 13% (n = 4) to the Environmental Factors component of the ICF. Six percent (n = 2) of the concerns did not link to the ICF. Of the 32 concerns identified by patients 14 mapped to the KOOS, 11 to the WOMAC and 4 to the Oxford Knee Scale.

**Conclusion:**

Patient concerns linked to four different components of the ICF indicating that patients are involved in or are thinking of multiple aspects of life even in this early phase of recovery. The KOOS was found to be the most appropriate for use based on the patients' perspective. However, less than half of the concerns identified by patients were covered by the KOOS, WOMAC or Oxford Knee Scale indicating that other existing measures that evaluate the concepts identified as important to patients should be considered when evaluating outcomes during this acute phase of recovery following primary TKA.

## Background

Knee osteoarthritis (OA) is a leading cause of disability in the elderly population [1]. Total knee arthroplasty (TKA) has been shown to be an effective procedure in reducing pain and improving function and quality of life in individuals suffering from OA of the knee [2,3]. While the initial emphasis of outcome measures was on clinical results that were important to physicians or surgeons [4], the trend has changed to measures that evaluate multiple domains, such as pain, physical function, health related quality of life and patient satisfaction [4-6]. In evaluating the success of any treatment, surgical or not, opinions of patients are of great significance [7]. Therefore, outcome measures used to measure change in a TKA population should have sound measurement properties, including content that reflects patient concerns during their rehabilitation.

Despite recognizing the importance of patients' perspectives in evaluating outcomes, published research on what is important to patients in the acute post-operative phase following primary TKA is scarce. Trousdale et al. [8] and Moran et al. [9] have both reported patient concerns prior to total hip arthroplasty (THA) and TKA. However, these authors do not report if the items of concern in their respective questionnaires were generated by directly asking patients. Weiss et al. [10] investigated what functional activities were important to patients one year after TKA surgery. Based on their findings they have suggested, "that conventional questionnaires and scoring systems may be missing important information about knee function" in patients who have had a joint replacement [10].

Even though the importance of the patients' perspective in choosing outcomes and in developing outcome measures has been recognized, the choice of outcomes is rarely grounded in a conceptual framework [11,12]. In recent years the International Classification of Functioning, Disability and Health (ICF) [13] has been widely adopted as a conceptual model for describing outcome measures. Briefly, the ICF is a biopsychosocial model of health that focuses on the consequences of disease (as opposed to a medical model focused on disease) and includes two parts, with each part containing separate components. The first part covers functioning and disability and includes the components of Body Structure and Function, Activities, and Participation. The second part covers contextual factors and includes the components of Environmental Factors and Personal Factors. The ICF provides a unified framework for evaluating health and health-related states of populations in both clinical practice and research [14].

Although a few studies have been done to determine what is important to individuals following TKA, no one has investigated what concerns patients may have in the acute post-operative phase (0–6 weeks) following primary TKA. Furthermore, patient concerns have not been linked to a conceptual model such as the ICF. Also, it is not known if patient concerns in the acute post-operative phase are in fact being measured by conventional outcome measures.

The objectives of this study were to: 1) identify what patients believe is important during their recovery in the first six weeks following primary TKA (i.e. their concerns), 2) link these concerns to components of the ICF; and, 3) map these concerns to items in three commonly-used self-report outcome measures.

## Methods

### Participants

Subjects were recruited from the inpatient orthopaedic ward and from the outpatient physiotherapy department at London Health Sciences Centre, University Hospital (LHSC-UH) in London, Ontario, Canada from February to May 2005. All subjects were patients of five orthopaedic surgeons working at the hospital at the time of the study. To be included, subjects must have had a primary TKA due to a diagnosis of OA of the knee, be in week 1–6 post-primary TKA, and be able to ambulate with or without gait aids. Subjects were excluded if they were unable to understand written or spoken English or were unable to provide informed consent. Thirty subjects were recruited to maximize the chances of reaching data saturation with the interviews and also to provide a balanced number of subjects (n = 5) in each one of the six post-operative weeks. The University of Western Ontario Research Ethics Board for Health Sciences Research Involving Human Subjects approved this study.

### Data collection tools and procedures

All interviews were conducted in person while the participant was an inpatient or during an outpatient physiotherapy session. Participants provided demographic information (age and gender) and were requested to answer the following question verbally by the primary investigator (RR). "What is important to you right now with respect to your recovery from knee replacement surgery?" During the interview, the primary investigator emphasized to every participant that s/he should only identify what was important to him or her during that particular week of the interview. The participants' answers were recorded in writing by the primary investigator. Interview duration was not controlled and varied from 5–15 minutes.

### Analysis

Subjects' answers were grouped on the basis of common themes. From these groupings, items of importance were created by paraphrasing the grouped responses. For example, items such as 'going down stairs', 'getting up and down a few stairs', 'to walk up/down a flight of stairs', 'going up and down stairs safely' and 'to get better at going up/down stairs' were paraphrased to two items or concerns, 'descending stairs' and 'ascending stairs'. From this point on we refer to the items of importance as patient concerns.

All of the concerns were linked to the components of the ICF independently by two of the authors (RR and AMD) using the operational definitions of the different ICF components as defined by the World Health Organization [13]. Body Structures have been defined as anatomical parts of the body and Body Function refers to physiological functions of the body systems including psychological functions. Activity has been defined as the execution of a task by an individual and Participation has been defined as involvement in a life situation that would likely involve other individuals. Environmental Factors include external factors in the life of an individual and Personal Factors are features of an individual such as age, gender, ethnic background, acquired habits and other personal characteristics unique to the individual. Environmental factors and Personal contextual factors may facilitate or hinder performance across ICF components. Any discordant linking between the two authors was discussed to achieve consensus.

The patient concerns were then mapped by the primary investigator to the Knee Injury and Osteoarthritis Outcome Score (KOOS) [15], the Western Ontario and McMaster Universities Osteoarthritis Index (WOMAC) [16], and the Oxford Knee Scale [17]. These three self-report measures were chosen because they have good psychometric properties, have been validated for use following TKA and are commonly used internationally to assess patient perceived health status following primary TKA [17-19]. The mapping was conducted by comparing the content of each paraphrased patient concern to the content of each item on the outcome measures mentioned above. A patient concern was considered to map to an outcome measure item if the content of the concern was the same as the content of an item in the outcome measure.

## Results

Of 31 subjects who were contacted, only one person refused to participate leaving 18 females and 12 males who were interviewed. Their average age (SD) was 68.4 (11.1) years. Five subjects were recruited in each of the six post-operative weeks except for post-operative week two and week five, where four and six subjects were recruited respectively.

After paraphrasing all interview responses into common themes, 32 different concerns were identified as being important to subjects with respect to their recovery in the first six weeks following primary TKA. Table 1 shows these concerns as a function of the post-operative week in which the concern was identified. The table shows that even though different people were interviewed within each post-operative week, certain concerns were identified earlier in the recovery process, some were identified later, while others were identified throughout the entire six-week time frame. For example, 'avoiding an infection' was identified as a concern in the first week of recovery, 'going back to your regular exercise class/sport' was identified in the fifth and sixth post-operative week and 'decreasing pain' and 'increasing the bend in the surgical knee' were identified in each one of the first six weeks following surgery.

**Table 1:** Patient concerns within six weeks following TKA^a ^(n = 30).

	**Post-operative week**					
**Concern**	**1**	**2**	**3**	**4**	**5**	**6**

Receiving competent care from health care workers in a timely manner	x					
Avoiding infection in your surgical knee	x					
Getting out of bed on your own	x					
Doing your exercises as prescribed by your physiotherapist	x	x	x	x		
Walking on flat surface	x	x	x	x	x	
Increasing the bend in your surgical knee	x	x	x	x	x	x
Decreasing pain in your surgical knee	x	x	x	x	x	x
Comfortably sit in car		x	x			
Getting in/out of bath		x		x		
Doing your own housework		x	x	x	x	
Walking on uneven ground		x	x	x	x	
Descending stairs		x	x	x	x	x
Cooking your own meals		x	x	x		x
Ascending stairs		x	x		x	x
Sleeping better at night		x		x	x	x
Light domestic duties		x		x	x	
Being independent		x				x
Getting in/out of car		x				x
Putting on your own shoes or socks			x			
Having the support of your family members			x			
Having the support of your neighbours			x			
Dressing yourself			x	x		
Being less of a burden on spouse or caregiver			x		x	
Receiving appropriate explanation regarding what to expect with rehabilitation following your surgery			x	x		x
Increasing the strength in your legs			x		x	x
Increasing the straightening in your surgical knee			x	x	x	x
Returning to your hobbies			x	x	x	x
Reducing swelling in your surgical leg				x		
Heavy domestic duties				x		
Going shopping				x	x	x
Driving a vehicle				x	x	x
Going back to regular exercise class/sport					x	x

^a ^TKA = primary Total Knee Arthroplasty

Note: the above concerns were ordered to illustrate the point in time when the concern was identified and x = at least 1 patient reported the concern

Table 2 presents the patient concerns grouped by their corresponding ICF component. Twenty-two percent (n = 7) linked to the Body Structure and Function component, 47% (n = 15) to the Activity component, 13% (n = 4) to the Participation component and another 13% to the Environmental Factors component. The concern, 'being less of a burden on your spouse or caregiver' was the only concern that was linked to two different components (Participation and Environmental Factors) by the two different authors. After discussion it was concluded that this concern best mapped to the Environmental Factors component of the ICF. Two concerns (6%), 'receiving appropriate information regarding what to expect with rehabilitation following your surgery' and 'being independent' could not be mapped to the ICF components.

**Table 2:** Patient concerns within six weeks following TKA^a ^by ICF^b ^and outcome measure (n = 30).

**Concern**	**KOOS^c^**	**WOMAC^d^**	**Oxford^e^**
**Body Function (7)**			
1. Decreasing pain in your surgical knee...............	P1-P9*	P1-5*	1*
2. Reducing the swelling in your surgical leg.........	S1		
3. Avoiding infection in your surgical knee			
4. Sleeping better at night			
5. Increasing the bend in your surgical knee..........	S5		
6. Increasing the straightening in your surgical knee	S4		
7. Increasing the strength in your legs			
**Activities (15)**			
1. Getting out of bed on your own......................	A10	F17	
2. Getting in/out of bath..................................	A13	F20	
3. Putting on your own shoes or socks.................	A9	F16	
4. Dressing yourself			
5. Walking on a flat surface.............................	A6	F13	
6. Walking on uneven ground			
7. Descending stairs......................................	A1	F8	12
8. Ascending stairs.......................................	A2	F9	
9. Cooking your own meals			
10. Doing your own housework			
11. Heavy domestic duties.................................	A16	F23	
12. Light domestic duties..................................	A17	F24	
13. Getting in/out of car....................................	A7	F14	3
14. Comfortably sit in car			
15. Doing your exercises as prescribed by your physiotherapist			
**Participation (4)**			
1. Driving a vehicle			
2. Going shopping........................................	A8	F15	11
3. Returning to hobbies (e.g. dancing, gardening)			
4. Going back to regular exercise class or sport			
**Environmental factors (4)**			
1. Being less of a burden on spouse or caregiver			
2. Having the support of your family members			
3. Having the support of your neighbours			
4. Receiving competent care from health care workers in a timely manner			

**Total number of concerns (32)**	14	11	4

^a ^TKA = primary Total Knee Arthroplasty

^b ^ICF = International Classification of Functioning, Disability, and Health

^c ^KOOS = Knee Injury and Osteoarthritis Outcome score (P, S, A = pain, symptom, activities of daily living subscale, respectively)

^d ^WOMAC = Western Ontario and McMaster Universities Osteoarthritis Index (P, F = pain, function subscale, respectively)

^e ^Oxford = Oxford Knee Scale

* Numbers in these columns refer to the item number in the original questionnaire

Note: Two concerns 'Receiving appropriate information regarding what to expect with rehabilitation following surgery' and 'Being independent' not covered by ICF & did not map to outcome measures

Table 2 also shows the mapping of patient concerns to three commonly used outcome measures following TKA. Of the three measures, the KOOS covered 14 patient concerns, the WOMAC covered 11 patient concerns while the Oxford Knee Scale covered four patient concerns.

## Discussion

This study has identified 32 concerns that are important to patients during their recovery in the first six weeks following primary TKA. All but two of the identified concerns mapped to four components of the ICF model. Approximately half of the patient concerns identified in this early phase of recovery were not addressed in the content of three commonly used outcome measures.

Two groups of researchers have independently documented concerns of patients prior to undergoing total hip and total knee arthroplasty [8,9]. In both of these studies, patients were asked to rate their concerns for a 54-item and 29-item questionnaire, respectively. Of the 54 concerns reported by Trousdale et al. [8], 13 concerns (pain, infection and stiffness in joint after surgery, walking, up and down stairs, in/out of bathtub, driving, dressing, ability to do usual work, return to recreational activities, not knowing what to expect and quality of therapy after surgery) were similar to the concerns identified by subjects in this study. Moran et al. [9] do not report all 29 items of the questionnaire used in their paper but reported the following top five patient concerns: cancellation of surgery, no decrease in pain, risk of losing the leg, risk of joint infection and risk of dying. While these concerns were generated for TKA and THA patients combined, at the time of this study there was no published evidence to suggest that concerns of patients in the early post-operative phase following total knee and hip arthroplasty are different. However, since that time researchers have shown that the level and time frame of recovery following total hip and knee arthroplasty is different [20,21], which may lead one to speculate that there may be some differences in concerns between these two patient populations. This may explain why only 13 concerns reported by Trousdale et al. [8] were similar to concerns in this study. Furthermore, concerns reported by Trousdale et al. [8] were generated prior to joint replacement surgery as opposed to early after surgery in our study.

Subjects interviewed in post-operative week one were still inpatients at a large tertiary care hospital. Based on the recovery process following primary TKA and the fact that these subjects were interviewed prior to discharge from acute care, it is apparent in Table 1 that patient concerns identified in the first week after surgery were related to this acute stage of their recovery. For example, concerns about competent care, avoiding infection, getting out of bed independently, doing your exercises and ambulation are all pertinent to the early post-operative time frame. Knee range of motion and specifically the amount of flexion is a commonly used outcome measure by health care professionals including surgeons following primary TKA [22]. Furthermore it is common for patients to experience pain following primary TKA [23]. At the time of this study, most patients following primary TKA at the study institution were discharged from hospital on post-operative day four or five. Accordingly, the majority of concerns identified by subjects from post-operative week two onwards (getting in/out of bath, doing housework, cooking, walking, getting in/out of car, comfortably sitting in car, driving, going shopping, returning to hobbies etc.) were activities that a person would normally perform while living in his/her own home.

Turning now to the ICF findings, it is revealing that even in this early phase of recovery following primary TKA, some patient concerns were linked to the ICF Participation component. For example, even within the first six weeks after surgery patients were thinking about 'driving a vehicle', 'going shopping', 'returning to hobbies' and 'going back to regular exercise class/sport'. The concerns about 'returning to sport' and 'driving a vehicle' are especially surprising considering that physical function deteriorates in the first month following TKA [21] and participants in this study were advised by the health care team not to drive for the first six weeks following surgery.

Concerns that did not map to the ICF were 'receiving appropriate information regarding what to expect with rehabilitation following your surgery' and 'being independent'. These concerns are consistent with previous research, which reported patient concerns about dependence [24] and the importance of education in the rehabilitation process following total joint replacement surgery [25].

In this study patient concerns were also mapped to the KOOS, WOMAC and Oxford Knee Scale. As shown in Table 2, 14 of the 32 patient concerns mapped to the KOOS, 11 to the WOMAC and four to the Oxford Knee Scale. With greater than half of all patient concerns missing from these commonly used outcome measures, this study highlights their lack of content validity when used in this early phase of recovery. However, this is not surprising as these outcome measures were not developed by evaluating patient concerns in this early phase of recovery and have traditionally been used by researchers to evaluate outcome a few months or years following TKA. To evaluate outcome in this early phase of recovery researchers and clinicians may need to use a combination of measures to capture the different components of the ICF (i.e. impairments of body structure and function, activity limitations, participation restrictions and environmental factors). Additionally, given the difference in content of currently used measures, such as the KOOS, WOMAC and Oxford Knee Scale and the content identified by patients as important in the early recovery period along with the suggestion from this work that patient concerns for recovery change over time, future research needs to consider outcome measurement in the context of how patient concerns for recovery change over time.

All participants in the study were recruited from a single tertiary care hospital. This may be viewed as decreasing the generalizability of this study to other settings. However, it should be noted that individuals undergoing TKA at this site were not only London residents but also travelled from communities across Southwestern Ontario including rural settings. Also, it is acknowledged that the setting of rehabilitation (home care, inpatient facilities, outpatient clinics) following surgery may influence what is important to patients. In this study, the majority of patients received therapy at home until post-operative week three. After this most patients continued therapy at an outpatient clinic of their choice. Therefore, concerns of patients receiving therapy in settings different from this study may or may not be the same. In addition, the presence or absence of a caregiver in the home may have influenced what patients felt was important to their recovery. Because the living status of patients was not measured, the effect of this variable on patient concerns in this study cannot be determined. Finally, it is possible that this study may not have captured all patient concerns within the first six weeks following primary TKA surgery. Another limitation of this study was that non-English speaking individuals were not included.

## Conclusion

In conclusion, this is the first study that has identified concerns that are important to patients in the first six weeks following their primary TKA. Using the ICF framework showed that patients are thinking of multiple aspects of life even in this acute phase of recovery. The KOOS, the WOMAC and the Oxford Knee Scale do not adequately measure patient concerns in the early phase of recovery as reported in this study. However, some of their content with well-tested items provides a starting point from which to develop measures that encompasses patient concerns for use in the early post-operative phase. Additionally, other existing measures that evaluate the concepts identified as important to patients should be considered in evaluating outcomes during this acute phase of recovery following primary TKA. Replication of the study results within the time frame of this study would strengthen the validity of our results. Also, further studies are required to identify if patient concerns in the long term post-operatively are being evaluated by commonly used self-report outcome measures.

## Competing interests

The author(s) declare that they have no competing interests.

## Authors' contributions

RR, AMD and BMC designed the study. RR collected and analyzed the data and drafted the manuscript with regular feedback from AMD and BMC. All authors read and approved the final manuscript.
